# Spatial variation and inconsistency between estimates of onset of muscle activation from EMG and ultrasound

**DOI:** 10.1038/srep42011

**Published:** 2017-02-08

**Authors:** Angela V. Dieterich, Alberto Botter, Taian Martins Vieira, Anneli Peolsson, Frank Petzke, Paul Davey, Deborah Falla

**Affiliations:** 1 Pain Clinic, Anaesthesiology, University Medical Center, Göttingen, Germany; 2 Laboratory of Engineering of the Neuromuscular System and Motor Rehabilitation, Department of Electronics, Politecnico di Torino, Torino, Italy; 3 School of Physical Education and Sports, Universidade Federal do Rio de Janeiro, Rio de Janeiro, Brazil; 4 Department of Medical and Health Sciences, Physiotherapy, Linköping University, Linköping, Sweden; 5 School of Physiotherapy and Exercise Science, Faculty of Health Sciences, Curtin University, Perth, Australia; 6 Centre of Precision Rehabilitation for Spinal Pain (CPR Spine), School of Sport, Exercise and Rehabilitation Sciences, College of Life and Environmental Sciences, University of Birmingham, Birmingham, UK

## Abstract

Delayed onset of muscle activation can be a descriptor of impaired motor control. Activation onset can be estimated from electromyography (EMG)-registered muscle excitation and from ultrasound-registered muscle motion, which enables non-invasive measurements in deep muscles. However, in voluntary activation, EMG- and ultrasound-detected activation onsets may not correspond. To evaluate this, ten healthy men performed isometric elbow flexion at 20% to 70% of their maximal force. Utilising a multi-channel electrode transparent to ultrasound, EMG and M(otion)-mode ultrasound were recorded simultaneously over the biceps brachii muscle. The time intervals between automated and visually estimated activation onsets were correlated with the regional variation of EMG and muscle motion onset, contraction level and speed. Automated and visual onsets indicated variable time intervals between EMG- and motion onset, median (interquartile range) 96 (121) ms and 48 (72) ms, respectively. In 17% (computed analysis) or 23% (visual analysis) of trials, motion onset was detected before local EMG onset. Multi-channel EMG and M-mode ultrasound revealed regional differences in activation onset, which decreased with higher contraction speed (Spearman ρ ≥ 0.45, P < 0.001). In voluntary activation the heterogeneous motor unit recruitment together with immediate motion transmission may explain the high variation of the time intervals between local EMG- and ultrasound-detected activation onset.

Delayed onset of muscle activation can be a clinically relevant descriptor of impaired motor control in painful conditions^1^,^2^,^3^. Activation onset is usually measured using electromyography (EMG), which registers, at the skin surface or through invasive electrodes, the change of electrical potential with muscle excitation. To non-invasively assess the onset of deep muscle activation, ultrasound measures of activation onset are increasingly utilised^4^,^5^,^6^. Ultrasound can be used to monitor muscle motion that occurs with muscle contraction^6^,^7^,^8^,^9^. Electrical muscle excitation initiates muscle contraction and is followed by a physiological electromechanical delay (EMD) before force production can be registered externally^10^,^11^,^12^,^13^. Using ultrafast ultrasound in electrically stimulated muscle, the EMD has been subdivided into a first phase from electrical stimulation until the ultrasound-measured onset of muscle fibre movement (4–7 ms), a second phase until the muscle’s tendon moves (0–2 ms), and a third phase until force production is registered externally (3–5 ms)^12^,^14^,^15^. These well-defined phases can be documented only in electrically stimulated activation^11^. In voluntary activation, the relationship between excitation and muscle motion onset is less clear. Variable time intervals of 20 ms^6^ up to **−**54 ms^9^ between EMG- and ultrasound-measured onset have been reported using M-mode or Doppler ultrasound, surface or fine-wire EMG. Moreover, all previous studies that compared EMG- and ultrasound-measured onset of voluntary activation, reported the detection of motion prior to EMG onset in 10% to 70% of the trials^6^,^7^,^8^,^9^,^16^. The majority of these trials were explainable by motion transmission from deeper, earlier activating muscle fibres^8^ or from adjacent, earlier activating muscles^6^,^9^. However, the mechanism behind approximately 20% of motion detection before EMG onset in deep, earlier activating muscles remained unexplained^6^,^7^,^9^. One potential influence is an imprecise technical synchronization of ultrasound and EMG data^6^,^17^,^18^. The uncertain relationship between EMG and motion onset in voluntary activation questions the use of ultrasound-detected motion onset of deep muscle activation to assess motor performance^4^,^5^.

As voluntary muscle recruitment is sequential^19^, may be inhomogeneous^11^,^20^ and may differ between muscle regions^21^,^22^,^23^, EMG and ultrasound signals should be sampled from the same muscle region for direct comparison. Recently, a multi-channel surface electrode was developed which is transparent to ultrasound thereby enabling EMG and ultrasound to be detected concurrently over the same muscle region^24^. Measurements of the onset of muscle motion require higher temporal acuity than that provided by conventional B-mode ultrasound. M(otion)-mode ultrasound enables a temporal resolution of 1–5 ms^25^ and permits the distinction of motion in superficial and deep muscle regions^7^,^26^,^27^. M-mode delineates the instantaneous positions of sound-reflecting tissue interfaces from a single ultrasound-beam which consecutively form a trace of displacement over time (Fig. 1). Muscle relaxation is indicated by the stable depth and greyscale of fascicular interfaces. Muscle contraction with fascicles moving through the ultrasound beam produces changes ranging from differing greyscale shade to rapidly alternating bright and dark tissue components^7^,^28^ (Fig. 1).

A range of different methods for estimating activation onset from EMG^29^,^30^,^31^,^32^ and ultrasound signals have been proposed^7^,^8^,^16^. In computed onset detection, the *onset* is usually defined by a difference from the relaxed baseline signal and therefore the validity of the measure is dependent on a relaxed, stable baseline of sufficient length^29^,^33^. The exact onset value depends on the predefined difference between relaxation and activation^32^,^33^. In visual onset detection, more complex signal characteristics are recognized^31^, but the subjective decision reduces the reliability of the measure.

In this study, we examined the temporal relation between EMG- and ultrasound-registered estimates of muscle activation onset, and factors that potentially influence the time interval between them. Specifically, the study objectives were to determine (1) the difference between EMG and motion onset when estimated from the same muscle region, (2) the earliest EMG onset estimated from multi-channel EMG and its relation to motion onset, and (3) the influences of the regional variation of EMG onset and of the contraction level and speed on the time interval between EMG and motion onset. Acknowledging the dependence of *onset* on the detection method, we performed a fully computed and a fully visual onset analysis, with the expectation that the exact time intervals between EMG and motion onset are method-dependent, but relations replicated by both analyses are likely valid. To avoid an influence of subjective decisions on onset variation, data exclusion was based on quantified criteria.

## Methods

Twelve healthy young men participated. Inclusion criterion was the subjective judgement of good general health. Exclusion criteria were trauma, surgery or skin irritability of the right arm, recurrent pain of the right arm, shoulder or neck, pain provoked by prolonged sitting and intake of medications that potentially affect reaction time. Two participants were excluded due to poor EMG data quality, thus the final sample included 10 men, aged 24.4 years (SD 3.3) with a BMI of 23.2 kg/m^2^ (SD 2.7). The study was approved by the ethics committee of the University Medical Center Goettingen (No. 25/6/14). Procedures were performed according to the Declaration of Helsinki. Participants provided written informed consent.

### Procedure

Multi-channel surface EMG (EMG-USB2, OTBioelettronica, Torino, Italy) and M-mode ultrasound (LogicScan 128, Telemed, Vilnius, Lithuania) were acquired concurrently as participants performed isometric flexion of their right elbow while seated upright in a Biodex System 3 (Biodex Medical Systems Inc., Shirley, NY, USA). The upper arm was supported with the shoulder positioned in 45° flexion and abduction, and externally rotated to expose the biceps brachii muscle. The elbow was positioned in 45° flexion and the handgrip in 40° of supination (Fig. 2a). Following a series of test contractions to familiarise the participant with the equipment, participants performed four maximal voluntary isometric contractions (MVIC) of elbow flexion, with verbal encouragement and 1-min rest between repetitions. The highest torque recorded over the four attempts was considered the MVIC to define the submaximal torque values. Following 5-min rest, participants performed two series of 10 trials of isometric elbow flexion. The two series included trials at 20%, 30%, 50% and 70% of MVIC such that each force level was performed five times. Trials were performed in randomized order with 25 s of rest after each contraction. A screen was positioned at a distance of ~1.2 m in front of the participant to provide visual feedback of the exerted torque and the target level (1.5 mm/Nm resolution). All participants received the same instruction to reach the target immediately and sustain the contraction over 5 s.

### High-density EMG

Differential EMG signals were collected using an innovative 32-channel (4 × 8) silicon rubber electrode (6 mm thick) transparent to ultrasound^24^, allowing EMG and ultrasound to be recorded concurrently from the same muscle region (Figs 2b and 3). The recording area was 3 cm × 7 cm. The recording cavities had a diameter of 4 mm and the interelectrode distance was 1 cm. The recording cavities of the electrode were filled with electrode cream (GE Medical Systems, Freiburg, Germany) and the grid was positioned over the mid biceps brachii with the lower border ~4 cm cranial to the elbow crease (Fig. 2b), following skin preparation. Reference electrodes were placed over the medial epicondyle and around the wrist. Signals were amplified (EMG-USB2, OTBioelettronica, Torino, Italy) by a factor of 500, collected at a sampling rate of 2048 Hz and were 12-bit analog-to-digital converted. Biodex torque data was sampled at 2048 Hz synchronously with the EMG.

### Ultrasonography

M-mode ultrasound data were collected using a linear transducer, 40 mm footprint (HL9/40), set to 9 MHz. The transducer was housed in a custom-shaped foam block, adapted to the curved surface of the muscle, and positioned in a perpendicular scanning angle. After fine adjustment of the transducer position and angle to achieve a clear image, the foam block was bandaged around the arm to secure the electrode and transducer positions (Fig. 2a). M-mode ultrasound was recorded in longitudinal orientation over a depth of 5 cm with a single focus at 3 cm, a dynamic range of 74 dB, a high contrast and highest sweep speed displaying 2.4 s, which resulted in a temporal resolution of 2 ms per vertical pixel line (505 pixel lines = 1000 ms). Notably, the temporal resolution of the M-mode trace is not indicated by the frame rate^25^,^34^. Similar to pulsed-wave Doppler, single scan lines are processed of which several are added to the trace with every frame.

### Data analysis

#### EMG signal processing

EMG and torque signals were processed using Matlab (R2013a, MathWorks, Massachusetts, USA). Raw EMG signals were zero-lag bandpass-filtered between 15–350 Hz and full-wave rectified for visual onset detection. For each EMG channel, the root mean-square (RMS) EMG amplitude was calculated and averaged over the initial 500 ms of baseline and over 2 s of sustained activation at the required force level. The signal to noise ratio (SNR) was computed as the ratio between the baseline level of EMG amplitude and EMG amplitude during the sustained activation. Trials were excluded when the baseline duration was <500 ms after the trigger. Signals with SNR <1.5, the threshold being the lowest in which tests indicated a visual detection of activation onset, were also excluded. Trials in which EMG baseline amplitude was >50% of the average baseline amplitude of all trials of the respective participant were discarded as they were considered as insufficient relaxation. Only trials in which a minimum of 50% of the EMG onset estimates were available were included in the analysis.

Automated EMG onset detection was performed with a previously proposed algorithm^35^. An occasional detection of EMG bursts prior to the main activation was accepted because an objective increase of EMG amplitude may elicit muscle motion and may explain the detection of early muscle motion. Visual onset was defined at the instant when a constant baseline pattern changed into a pattern of increasing amplitude. Visual detection was performed on longitudinal channel groups, blinded for previous EMG, muscle motion and torque onsets and was repeated by the same observer after 6–10 days to test intra-rater reliability. We determined (i) EMG onset averaged from the four channels directly surrounding the ultrasound beam (EMG_atbeam_) (Fig. 3), (ii) the earliest EMG onset (EMG_first_), (iii) the distance of the EMG_first_ channel from the location of the ultrasound beam in cm, and (iv) the standard deviation (SD) between EMG onsets across the 28 channels.

#### M-mode signal processing

**Figure 1:**
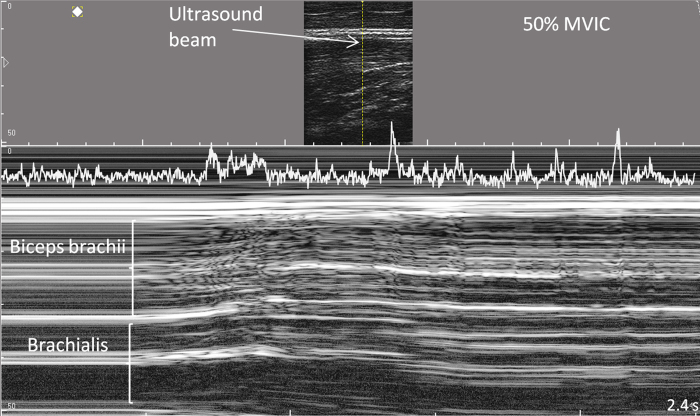
M-mode ultrasound trace of the biceps brachii and brachialis muscles during isometric elbow flexion at 50% of the maximal force. Note the gradually increasing motion that starts in the deeper muscle regions. The TKEO-transformation of the greyscale values of the upper cm of the biceps brachii is projected into the top of the trace.

**Figure 2:**
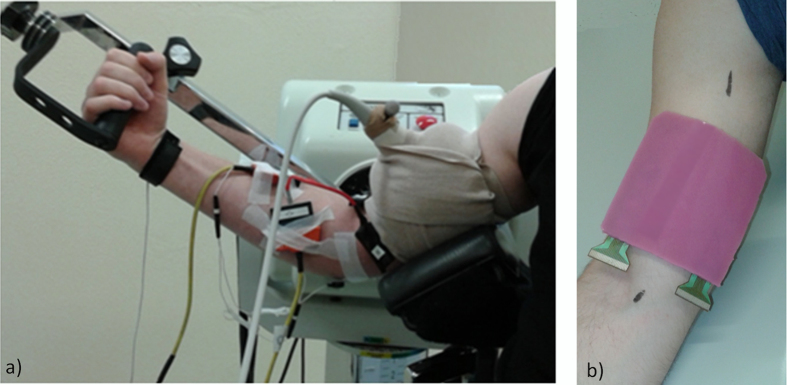
(**a**) Experimental setup, (**b**) electrode positioned over the biceps brachii.

**Figure 3:**
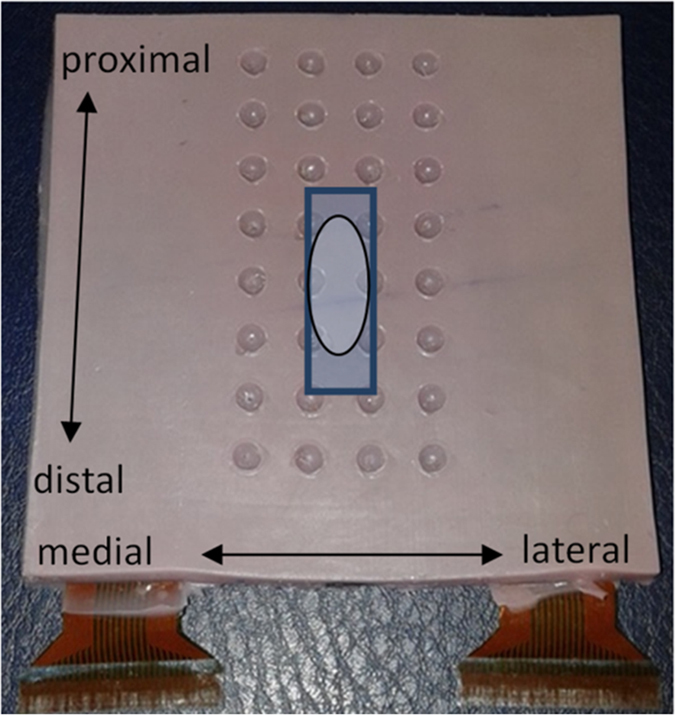
Multi-channel electrode transparent to ultrasound. The rectangle indicates the position of the ultrasound transducer whereas the oval indicates the electrode region which was compared as “at ultrasound beam”.

The greyscale values of each horizontal pixel line of the M-mode trace over the full depth of the biceps brachii muscle (Fig. 1) were retrieved using a custom LabVIEW application (2011 SP1, National Instruments, Texas, USA) and were transformed using the Teager-Kaiser Energy Operator (TKEO), TKEO = x^2^(n) − x(n − 1) * x(n + 1)^7^,^36^,^37^. The TKEO is an energy-detecting algorithm that enhances the detection of signal changes^38^. The TKEO-transformed signals were rectified and the mean TKEO amplitude of each sample of 2 ms was determined for the biceps brachii region directly underlying the surface electrodes (superficial 1 cm of muscle depth) and for the full biceps brachii (Fig. 1). Onset of muscle motion was computer-detected when the TKEO-transformed signals exceeded 2.5 SD of baseline TKEO amplitude, the threshold being determined in pilot tests. Visual onset of muscle motion was defined at the start of a continuing change in greyscale in more than three horizontal pixel lines and was measured using system in-built callipers. Visual onset detection was repeated for 50% of data after 2 weeks to test intra-rater reliability. Trials in which TKEO_baseline_ was >50% of the average baseline TKEO of the respective subject were considered insufficient relaxation and were discarded. Automated, baseline-dependent M-mode onsets cannot be compared between different depths because ultrasound noise increases with depth, leading to less sensitive onset detection in deeper muscle regions^27^. Therefore, superficial and deep motion onsets were compared only in the visual analysis. Ultrasound noise hindered automated measurements of the onset of brachialis muscle motion, which may influence biceps brachii motion. When brachialis motion was observed prior to biceps brachii motion, visual onset was determined. We determined (v) motion onset in the superficial biceps brachii (motion_sup_), (vi) motion onset over full biceps brachii depth (motion_deep_) and (vii) the visually detected difference between motion_sup_ and motion_deep_.

#### Torque signal processing

The automated detection of torque onset was defined when the torque amplitude exceeded 4 SD of baseline amplitude within the initial 500 ms. Visual torque onsets were defined at the starting point of a continuing increase of torque. Torque onsets served to determine contraction speed (rate of torque development, RTD), for the 200 ms following computed or visual torque onset, *RTD* = (*t*_*200ms*_ − *t*_0_)/(*0.2* *s*).

#### Data synchronization

The ultrasound system was equipped with a separate, analogue output channel that produced a TTL synchronization signal with the start of scanning. The TTL signal was collected by the EMG amplifier in a separate channel. EMG, torque and TTL signals were collected continuously over a contraction series and the ultrasound signal collection was started and stopped with each contraction.

The system-internal processing time of the raw ultrasound echoes may delay the start of the M-mode trace relative to the trigger signal^17^,^18^. The ultrasound processing time under study conditions was determined experimentally using a glass ball of 2.5 cm diameter falling onto a force plate with a water-filled latex glove (5 mm thick) that was attached to the ultrasound transducer. The impact of the ball falling from a height of 1.2 m created a sharp onset in both the M-mode and the force plate data. The comparison of the trigger and the M-mode signals in 50 repetitions revealed an unexpectedly large lag of the M-mode signals of 83.9 ms (SD 2.5). The measured ultrasound onsets were corrected by 84 ms to account for the lag.

### Statistical Analysis

Intra-rater reliability of visually determined EMG and muscle motion onsets was estimated by ICC (3, 1), mean difference and standard error of measurement (SEM) with ICC >0.75 being interpreted as good^39^. Time intervals between EMG and motion onset were skewed in distribution and required non-parametric descriptors, median and interquartile range (IQR). Differences between EMG_first_-motion and EMG_atbeam_-motion intervals were examined using the Wilcoxon signed ranks test. To investigate relations, skewness was reduced by the exclusion of extreme outliers, 7 trials (4.5%) in computed analysis and 6 trials (3.5%) in visual analysis. Potential factors of influence on the time interval between EMG and motion onset were related to (1) the regional variation of activation onset within the biceps brachii muscle (SD between onsets in the EMG channels, distance of EMG_first_ to the ultrasound beam, visual difference between motion_sup_ and motion_deep_) and to (2) the performance of muscle contraction (target force level, contraction speed). Correlations between EMG-motion time intervals and potential factors of influence were examined using Spearman rank correlation. Acknowledging the strongest influence, subsamples with opposing conditions were selected to clarify the influence on onset relationships. Significance was set to α = 0.05.

## Results

**Figure 4:**
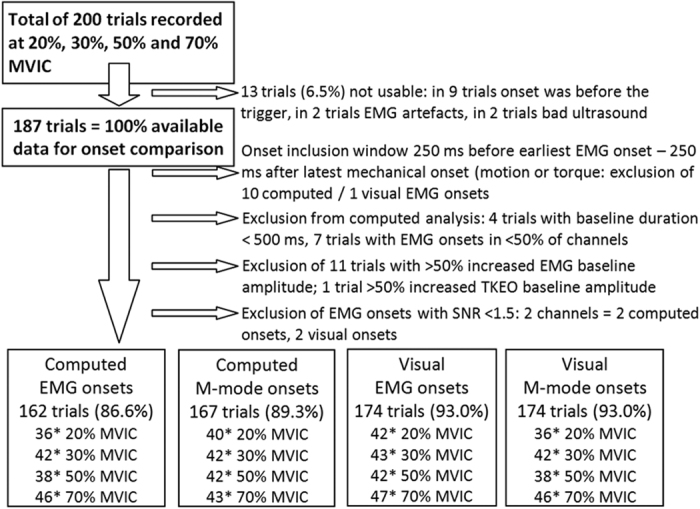
Data inclusion process.

A minimum of 87% of data files were included in the analyses (Fig. 4). The reliability of visual onsets was good^39^ (Table 1).

### Time intervals between EMG and muscle motion onset

**Figure 5:**
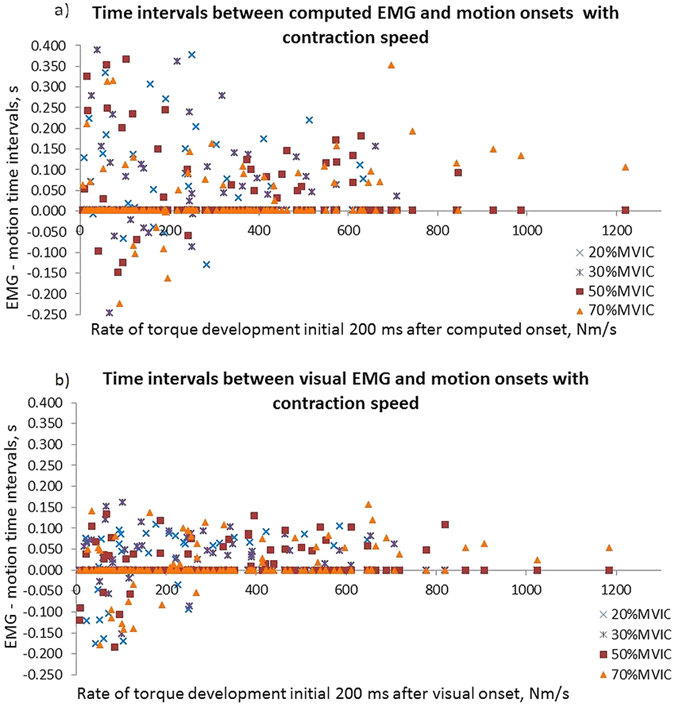
(**a,b**) Distributions of time intervals between EMG onset at the ultrasound beam and motion onset in the superficial muscle with contraction speed, in the different force levels. Negative time intervals indicate the detection of motion before EMG onset. Note the different distributions in the computed and the visual analysis and the reduction of negative time intervals with higher rate of torque development.

**Figure 6:**
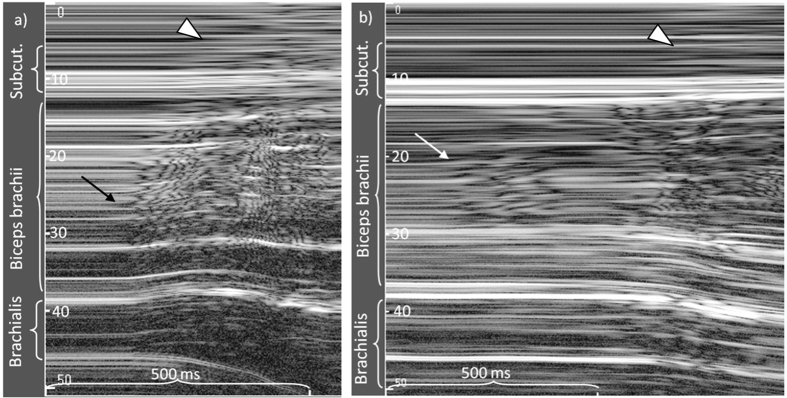
(**a,b**) Examples of regionally different muscle motion onset during voluntary isometric elbow flexion; motion started (**a**) earlier in the deep region of the biceps compared to the superficial region (54% of trials), (**b**) earlier in the mid-biceps region first (few trials of one participant). Earlier onset in superficial region of the biceps brachii relative to the deeper region was observed in 9% of trials, synchronous onset (difference <10 ms) in 35% of trials. Note the different appearance of motion by muscle bulging (arrowhead) in the region of the electrode (upper 6 mm) and the subcutaneous tissue.

The study results demonstrated large variation of the time intervals between EMG and motion onset (Fig. 5a,b). When comparing EMG and motion onset of the same muscle region, the computed EMG onsets were detected 96 ms (IQR: 121 ms) before motion onset and visual EMG onsets were detected 48 ms (IQR: 72 ms) before motion onset. The computed analysis resulted in larger variation and in 17% of the trials, motion detection occurred before EMG onset, versus in 23% of the trials in the visual analysis (Table 2 and Fig. 5a,b). In the trials in which motion was detected before EMG onset, motion was earlier by 68 ms (IQR: 84 ms) for the computed analysis and by 101 ms (IQR: 98 ms) for the visual analysis. Onset variation expressed as the SD between EMG channels was 73 ms (IQR: 77 ms) in the computed and 28 ms (IQR: 30 ms) in the visual analysis. The time interval between the first EMG onset and motion onset in the superficial muscle was 182 ms (IQR: 274 ms) for the computed analysis and 94 ms (IQR: 63 ms) for the visual analysis (Table 2). The M-mode ultrasound traces indicated differences in motion onset with muscle depth. The visually detected onset of muscle motion was earlier in the deep region of the biceps brachii in 55% of trials, with a median of 12 ms (IQR: 43 ms) compared to the superficial region (Figs 1 and 6).

### Influences on the time interval between EMG and motion onset

In both analyses, the EMG-motion time interval was significantly correlated with onset variation between EMG channels (computed: Spearman correlation coefficient rho (ρ) > 0.263, P < 0.001; visual: ρ > −0.297, P < 0.001), and with the variation of motion onset between the superficial and the deep muscle (visual analysis: ρ = −0.443, P < 0.001) (Tables 3, 4 and Fig. 5a,b). In both analyses, the distance of the first detected EMG onset to the ultrasound measurement location had no influence on the EMG-motion time intervals (ρ < 0.132, P > 0.103). Also the force level had no influence (ρ < 0.061, P > 0.435). Higher rate of torque development reduced the EMG-motion intervals (visual analysis: ρ = 0.328, P > 0.001) (Table 4 and Fig. 5b). In the computed analysis, the reduction of positive and negative time intervals precluded a description by correlation (Fig. 5a). In both analyses, the strongest correlations were between contraction speed and regional EMG onset variation (computed: ρ = −0.450; visual: ρ = −0.636, both P < 0.001) (Tables 3 and 4), which demonstrates reduced onset heterogeneity with increasing contraction speed. Brachialis motion was detected before deep biceps brachii motion in 7 trials. The influence of brachialis motion was inconsistent between the two analyses. Of the 7 trials, 0 trials for the computed analysis and 4 trials for the visual analysis demonstrated motion onset before EMG.

**Figure 7:**
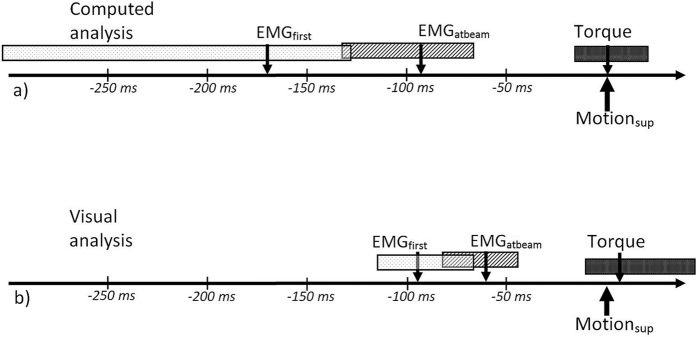
Onset relations in the 50 trials with the highest rate of torque development: timeline with sequence of onsets relative to muscle motion onset (=0 ms), arrows represent the median, boxes interquartile range; Abbreviations: EMG_first_, first EMG onset in multi-channel EMG; EMG_atbeam_, EMG onset in the channels at the location of the ultrasound; Motion_sup_, motion onset at the superficial biceps brachii muscle.

Comparing the 50 trials with the highest and the lowest contraction speeds, both analyses indicated larger EMG-motion time intervals and approximately four times higher variation with slow contraction speeds; the medians of the EMG-motion time intervals were 94 ms (IQR: 68 ms)/60 ms (IQR: 38 ms) in rapid contractions (Fig. 7) versus 108 ms (IQR: 251 ms)/35 ms (IQR: 167 ms) in slow contractions, for the computed/visual analysis, respectively. The detection of motion onset before EMG onset occurred in 29%/37% (computed/visual analysis) of the 50 trials with slow contraction speed, but in none of the 50 trials with higher contraction speed.

## Discussion

Both the computed and the visual onset analyses indicated a largely variable time interval between the onset of EMG-registered muscle excitation and ultrasound-detected muscle motion, which differed between sampling regions and which demonstrated an electromechanical delay in the majority of trials. EMG onset at the ultrasound beam was commonly not the first EMG onset. In approximately 20% of trials, both analyses detected muscle motion before local EMG onset. Earlier detection of EMG onset in channels away from the ultrasound beam and earlier motion detection in the deep biceps brachii compared to the superficial muscle region suggests muscle excitation started in variable regions of the biceps brachii including the deep muscle. Higher contraction speed reduced the regional variation of EMG onset and the variation of the time intervals between EMG and motion onset.

In the current study the regional variation of EMG onset in voluntary biceps brachii activation was larger than reported by Hug and colleagues^11^ which is explainable by the slightly different tasks. In the study of Hug the subjects were asked to contract as fast as possible. In the current study, the instruction to reach immediately a defined target force level resulted in a variable rate of torque development. Understandably, regional onset variation was higher with slower contraction speed when sequential motor unit recruitment was distributed over a larger time span. With slower contraction speed, the discrepancies between EMG and motion onset became more obvious. The early detection of motion during slow contractions cannot be explained by local muscle excitation and requires the consideration of other sources of muscle motion.

The detection of muscle motion before local EMG onset in approximately 20% of trials (17% for the computed and 23% for the visual analysis) is in agreement with earlier reported results^6^,^7^,^8^,^9^. Determination of the time interval between the *first* detected EMG onset and motion onset reduced trials with early muscle motion by 14% in the computed analysis and by 11% in the visual analysis. This suggests that the first activating motor units elicited muscle motion that was transmitted through the biceps brachii concurrently with ongoing recruitment. Although the sampling region influenced the EMG-motion time interval, the distance of the first detected EMG onset to the location of the ultrasound beam had no significant influence. Possibly, the first detected EMG onset at the surface was not the myoelectric onset that initiated muscle activation. M-mode ultrasound indicated that muscle motion started often in the deep biceps brachii region, which suggests an initial muscle excitation in the deeper muscle region. Differential activation between deep and superficial biceps brachii regions has been reported by a fine-wire EMG study^40^ and has its structural basis in six neuromuscular compartments which are innervated from the deep aspect of the biceps brachii^41^. The inter-electrode distance of 1 centimetre between the EMG channels suggests the detection volume was limited to the superficial biceps brachii^42^. Sequential recruitment is expected to follow the size principle and start with low-threshold motor units^19^. The deep biceps brachii contains a larger proportion of low-threshold slow twitch muscle fibres than the superficial region of the muscle^42^,^43^. A myoelectrical onset in a muscle region outside of the detection area of the surface electrode together with immediate force transmission may explain the observation of motion prior to local EMG onset or even prior to the first detected EMG onset at the surface.

The mechanism of ‘lateral force transmission’^44^ potentially explains early motion that has not been elicited by adjacent motor units at the muscle surface. While the longitudinal transmission of forces through muscle is thought to need time to stretch the series of elastic elements^12^, lateral force transmission through translaminar shear linkage has been described as being immediate^45^,^46^. Considering a sequential motor unit recruitment^19^ which may vary between muscle regions^20^, and intermingled muscle fibres from different motor units^47^,^48^, translaminar shear is expected to occur between the endomysium of already contracting and later or lesser contracting muscle fibres. Lateral force transmission is thought to bridge fibres that activate later or lesser and distribute intramuscular forces to maintain fibre alignment and the muscle’s integrity^45^,^49^,^50^. In an *in-vivo* muscle preparation, lateral force transmission was strong enough to transmit more than 50% of total muscle force in the tibialis anterior^51^. Usually, lateral force transmission is measured by its effects, i.e. the transmitted forces^51^,^52^,^53^,^54^. Understanding the onset of muscle motion as the first recognizable stage of muscle-internal force transmission^12^, ultrasound opens new options for investigation.

The time intervals between EMG and motion onset differed between automatic and visual detection, which reflects different onset definitions and the method-dependence of the precise instant of onset. The distributions of automated and visual time intervals (Fig. 5) reflect the different characteristics of each detection method. Automated onsets were highly sensitive to early EMG bursts which caused long EMG-motion time intervals (Fig. 5a). When the baseline included such bursts or higher noise, automated EMG onsets were later detected, which raises the question whether the detection of EMG after motion was caused by higher EMG baseline amplitude. Spearman correlation indicated no significant influence of EMG baseline amplitude on the EMG-motion time interval, ρ < 0.050, P > 0.534. Our definition of visual onset required a definite increase of EMG signal amplitude and ignored initial bursts, which led to a less sensitive detection of visual EMG onsets. The two analyses reflect the uncertainty and method-dependency of onset detection in voluntary muscle contractions; they may represent two poles of possible results, that would in detail differ with other computed settings, other automated detection methods or other visual raters^32^,^33^. The main study results were replicated with both analyses and do likely not depend on the detection methods.

Although this study measured regional muscle activation over a larger muscle region than previous studies which compared EMG- and ultrasound-detected activation onset, the study is limited to EMG detection at the muscle surface and a single ultrasound view into the muscle’s depth. The activation of motor units in deeper regions of the muscle could not be demonstrated. Technically this is not feasible, although an extension of the small sampling volume of fine-wire electrodes has been achieved^55^. Not all muscle motion must have originated in muscle contraction. Other possible sources of motion would be the relaxation of antagonist muscles, the transmission of stabilizing activity of scapular muscles or of thorax and shoulder motion. The following arguments support that the observed motion did mainly reflect muscle contraction. Motion transmitted from other body regions would likely affect the full biceps muscle, and would not produce a visible differentiation of motion onset between superficial and deep muscle regions (Figs 1 and 6). The smooth transition from slight motion (greyscale change of interfaces that maintain their depth) to motion typically associated to muscle excitation (rapid change of hyperechoic and hypoechoic interfaces^7^ (Fig. 1). Suggests an intramuscular process. Further, motion from preparing or bracing activities is expected to be more pronounced with higher force levels than with 20% or 30% MVIC, but there was no difference, not even a trend, between force levels. Although we attempted carefully to minimize movement of the body during the experiment, a contribution of motion from other sources cannot be excluded and will always be present in clinical measurement situations.

Notably, the synchronization experiment indicated that inconsistencies of the ultrasound processing time (SD 2.5 ms, range 11 ms) contributed to the variation of the EMG-motion time intervals. Relative to the overall variation of the EMG-motion intervals (Fig. 5) the influence of the variation of the synchronisation bias was very small, i.e. 2.1% for the computed analysis and 3.5% for the visual analysis. The results of the synchronization experiment demonstrated unexpected limitations of a current ultrasound system for highly precise synchronization. This emphasizes the current technical limitations of precisely relating ultrasound-measured mechanical events during muscle activation to external data and the importance of a verification of the data synchronization^17^,^18^.

During the initial check of data quality, the EMG data of two participants were excluded due to increased baseline noise levels, which may be due to a suboptimal handling of the new silicon electrode. The subsequent exclusion by quantitative criteria may have excluded more data and may have included more outliers than a procedure by visual inspection. Considering an inclusion >86% our procedure appears to be justified for a reproducible approach and results confirmed by two different onset detection methods. However, this revealed an unexpectedly high variation between local muscle excitation and contractile motion.

In conclusion, inconsistent time intervals between EMG- and ultrasound measured onset of voluntary muscle activation can be explained by a regional variation of activation onset, by methodological imperfection and by the different pathways of myoelectrical and myomechanical transmission, i.e. immediate intramuscular force transmission during ongoing motor unit recruitment. Local EMG onset may not represent the true onset of muscle activation and it does not determine the onset of muscle motion. The consistency of EMG- and ultrasound measured activation onset is improved in fast muscle contractions. Muscle motion onset marks a myomechanical activation onset that may be a useful, non-invasively measurable parameter of muscle activation.

## Additional Information

**How to cite this article**: Dieterich, A. V. *et al*. Spatial variation and inconsistency between estimates of onset of muscle activation from EMG and ultrasound. *Sci. Rep.* **7**, 42011; doi: 10.1038/srep42011 (2017).

**Publisher's note:** Springer Nature remains neutral with regard to jurisdictional claims in published maps and institutional affiliations.

## Figures and Tables

**Table 1:** Reliability of visually determined activation onsets from EMG and M-mode ultrasound signals.

	ICC_3,1_ (C.I.)	Range of ICCs of the 28 EMG channels	Difference (SEM) between rating sessions
EMG	0.992 (0.988–0.995)	0.982–0.996	1 ms (59 ms)
Motion_sup_	0.997 (0.995–0.998)		4 ms (44 ms)
Motion_deep_	0.998 (0.997–0.999)		3 ms (33 ms)

For the EMG data, the average across the 28 channels is given.

**Table 2:** Description of the distributions of the time intervals between EMG, muscle motion and torque onset, comparing EMG onset in the channels surrounding the ultrasound beam (EMG_atbeam_) and the earliest detected EMG onset (EMG_first_), and motion in the superficial biceps brachii muscle (motion_sup_) and motion over full biceps depth (motion_deep_).

	Computed analysis	Visual analysis
EMG_atbeam_		
Motion_sup_	96 ms (42–163 ms)	48 ms (7–79 ms)
	17% motion before EMG	23% motion before EMG
Motion_deep_	101 ms (53–188 ms)	32 ms (−46–67 ms)
	17% motion before EMG	38% motion before EMG
Torque	53 ms (−82–111 ms)	55 ms (14–89 ms)
EMG_first_		
Motion_sup_	182 ms (108–382 ms)	94 ms (60–123 ms)
	3% motion before EMG	12% motion before EMG
Motion_deep_	202 ms (107–403 ms)	76 ms (17–108 ms)
	3% motion before EMG	22% motion before EMG
Torque	136 ms (56–295 ms)	99 ms (64–130 ms)

Presented as median (lower quartile–upper quartile). Negative values indicate a detection of motion before EMG onset. Further, the percentage of trials in which motion has been detected earlier than EMG is given.

**Table 3:** Computed analysis, bivariate relations of the EMG-motion time interval and relevant factors of influence by Spearman rank correlation (ρ); significant correlations marked, *P < 0.05, **P < 0.001.

	EMG_atbeam_–motion_sup_	EMG_atbeam_–motion_deep_	EMG between-channels SD
EMG between-channels SD	ρ = 0.278**P < 0.001	ρ = −0.263**P < 0.001	
Rate of torque development	ρ = −0.024 P = 0.766	ρ = −0.069 P = 0.390	ρ = −0.450**P < 0.001

**Table 4:** Visual analysis, bivariate relations of the EMG-motion time interval and relevant factors of influence by Spearman rank correlation (ρ); significant correlations marked, *P < 0.05, **P < 0.001.

b) Visual analysis	EMG_atbeam_–motion_sup_	EMG_atbeam_–motion_deep_	EMG between-channels SD	Motion depth differences
EMG between-channels SD	ρ = −0.297**P < 0.001	ρ = −0.337**P < 0.001		
Motion depth differences	ρ = −0.073 P = 0.319	ρ = −0.443**P < 0.001	ρ = −0.223**P = 0.002	
Rate of torque development	ρ = −0.258**P < 0.001	ρ = −0.328**P < 0.001	ρ = −0.636**P < 0.001	ρ = −0.170*P = 0.021

Note that M-mode onset differences with muscle depth were only determinable in the visual analysis.
